# A rare case of acute urinary retention due to hematocolpos in a 15-year-old girl: A case report

**DOI:** 10.1016/j.ijscr.2024.110780

**Published:** 2024-12-24

**Authors:** Roland Muyisa, Emile Watumwa, Elisabeth Mathe, Moise Ndungo, Jean-Louis Muyisa

**Affiliations:** a Medicine, Catholic University of the Graben, Butembo, North Kivu, Democratic Republic of the Congo; b General Referral Hospital of Musienene, Territory of Lubero, North Kivu, Democratic Republic of the Congo; c Pharmacy, Catholic University of the Graben, Butembo, North Kivu, Democratic Republic of the Congo

**Keywords:** Acute urinary retention, Hematocolpos, Teenager

## Abstract

**Introduction and importance:**

Acute urinary retention (AUR) is uncommon in pediatric and adolescent populations, particularly among females.

To highlight the presentation of AUR as a symptom of hematocolpos due to an imperforate hymen in a 15-year-old girl.

**Case presentation:**

A 15-year-old girl presented with AUR and lower abdominal pain, which led to the diagnosis of hematocolpos.

**Clinical discussion:**

This case underscores clinicians' need to consider gynecological conditions in adolescent females with urinary retention. Early diagnosis and prompt surgical intervention are crucial to prevent complications, such as hydronephrosis and renal damage.

**Conclusion:**

Clinicians should have a heightened awareness of this rare but significant cause of AUR, particularly in patients with a history of primary amenorrhea and cyclic abdominal pain.

## Highlights

• In this cases report, we discuss the presentation of a 15-year-old girl who presented with acute urinary retention as the primary symptom of hematocolpos due to the imperforate hymen.
• This case reinforces the need for a high index of suspicion for gynecological conditions like hematocolpos in adolescent females presenting with urinary retention.
• Early diagnosis and prompt surgical intervention are crucial to prevent complications such as hydronephrosis and renal damage.
• Clinicians should be aware of this rare but significant cause of AUR, particularly in patients with a history of primary amenorrhea and cyclic abdominal pain.

## 1 Introduction

Acute urinary retention (AUR) is a relatively uncommon condition in the pediatric and adolescent populations, especially among females. While AUR is frequently encountered in adult males, particularly those with benign prostatic hyperplasia, its occurrence in young females often indicates a broader and more complex differential diagnosis [1]. The causes of urinary retention in adolescent females can range from neurologic disorders and infections to structural anomalies, with hematocolpos being a rare but important consideration [2].

In this case report, we discuss the presentation of a 15-year-old girl who presented with acute urinary retention as the primary symptom of hematocolpos due to the imperforate hymen. This underscores the need for clinicians to consider gynecological causes in adolescent females with AUR and highlights the importance of timely intervention to prevent further complications.

## 2 Case presentation

A 15-year-old previously healthy female adolescent, identifying as Christian and attending secondary school, presented to the emergency department with a two-day history of acute urinary retention and five days of progressively worsening lower abdominal pain. She denied fever, vomiting, or recent trauma. The patient had experienced several similar episodes of mild abdominal discomfort over the past few months, particularly around the time when she believed her menstrual periods should have started. However, she had never experienced any menstrual bleeding (primary amenorrhea).

The patient reported a 13-month history of cyclic abdominal cramping, initially attributed to delayed menarche and managed with indigenous medicines. Her mother reported that the patient had developed normal secondary sexual characteristics at the expected age (thelarche at 12 years, pubarche at 13 years). Still, no menstrual period had occurred despite the patient's development being otherwise normal.

The patient had no known medical conditions, and her family history was unremarkable for any gynecological or urinary issues. She had not been sexually active and denied any history of infections or pelvic surgery. There were no symptoms of constipation, changes in bowel habits, or abnormal vaginal discharge.

### 2.1 Physical examination

On physical examination, the patient appeared anxious and uncomfortable due to abdominal pain. Her vital signs were within normal limits: blood pressure 112/70 mmHg, heart rate 84 beats per minute, respiratory rate 18 beats per minute, and a temperature of 36.8 °C.

Abdominal examination revealed a distended lower abdomen with tenderness on palpation, particularly in the suprapubic region. No organomegaly was noted.

A focused genitourinary examination, performed after obtaining consent, revealed a bulging, tense, bluish hymenal membrane consistent with an imperforate hymen. No external vaginal bleeding was visible, and there were no signs of trauma or infection. Rectal examination revealed a firm, tender mass behind the hymen, suggestive of fluid accumulation in the vaginal canal.

### 2.2 Diagnosis workup

**Fig. 1:**
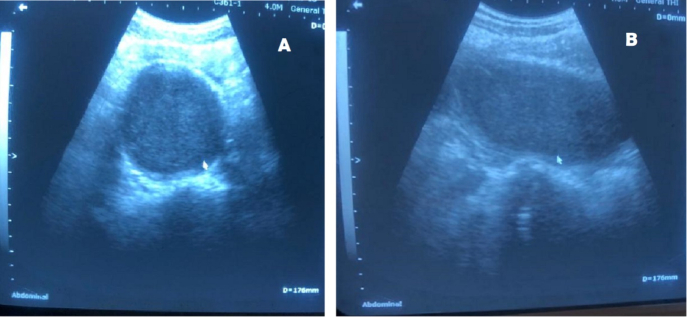
Abdominal ultrasound (A and B: vaginal cavity with hemorrhagic content after bladder catheterization).

Given the findings, an abdominal ultrasound was promptly performed to confirm the suspected diagnosis. The ultrasound demonstrated a markedly distended vagina and uterus, filled with hypoechoic fluid consistent with blood, confirming the presence of hematocolpos (Fig. 1). The uterus appeared enlarged, with no other structural abnormalities noted. There was mild bilateral hydronephrosis, likely secondary to the pressure exerted by the distended uterus and vaginal canal on the bladder and ureters.

Laboratory evaluations were conducted, yielding results that were largely within normal limits. The complete blood count (CBC) displayed findings consistent with normal physiology, with parameters indicating a color range from pale yellow to amber and a clear appearance, all falling within the established normative range of 4.5 to 8.0. Urinalysis results further supported the absence of any pathological conditions, showing negative results for glucose and ketones, with minimal trace amounts of proteins noted, alongside negative findings for nitrites and leukocyte esterase. Additionally, urinary sediment analysis revealed a count of 0–2 red blood cells and 0–5 white blood cells per high power field (HPF), with no evidence of bacterial presence. Serum creatinine and blood urea nitrogen (BUN) levels remained within established normal ranges, suggesting that there was no significant renal impairment despite the presence of hydronephrosis.

### 2.3 Diagnosis

The combination of the patient's clinical presentation, physical examination findings, and imaging results led to a diagnosis of hematocolpos secondary to an imperforate hymen, with acute urinary retention due to compression of the bladder outlet by the distended vaginal and uterine structures.

### 2.4 Management

The patient was admitted to the hospital for urgent surgical intervention. A hymenectomy was conducted under rachianesthesia to alleviate the obstruction and facilitate the drainage of accumulated hematoma. During the procedure, approximately 1200 mL of dark, viscous menstrual blood was drained from the vaginal cavity. No complications were encountered during the surgery.

Following the procedure, the patient experienced rapid relief of her urinary symptoms and abdominal pain. She was monitored overnight for any pod-operative complications and was discharged the following day in stable condition with instructions for follow-up.

### 2.5 Outcome and follow-up

At her two-week post-operative visit, the patient reported complete resolution of her urinary retention and abdominal discomfort. Her first normal menstrual period occurred four weeks after the procedure. A repeat pelvic ultrasound showed resolution of the hematocolpos and improvement of the previously noted hydronephrosis. The patient was referred to a gynecologist for ongoing care and education regarding her menstrual health.

## 3 Discussion

This case report presents a rare instance of acute urinary retention (AUR) secondary to hematocolpos in a 15-year-old girl with an imperforate hymen. The case underscores the importance of recognizing gynecological causes in the differential diagnosis of urinary retention in adolescent females, particularly when associated with primary amenorrhea and cyclic abdominal pain. While AUR is relatively common in males due to conditions such as benign prostatic hyperplasia, its occurrence in females, especially young girls, is unusual and often indicates a more complex underlying condition [1].

Hematocolpos, which results from the accumulation of menstrual blood in the vagina due to an obstruction such as an imperforate hymen, can exert significant pressure on surrounding pelvic structures, including the bladder and urethra. This mechanical pressure can lead to urinary retention, as seen in this case, where the distended vaginal and uterine cavities obstruct normal urinary outflow. Similar cases have been reported in the literature, where hematocolpos is associated with urinary symptoms such as urgency, frequency, or, more rarely, complete retention [3,4]. This highlights the need for a thorough clinical and gynecological assessment in adolescent girls presenting with urinary complaints.

The diagnosis of hematocolpos is often delayed due to the non-specific nature of early symptoms, such as lower abdominal pain and urinary difficulties, which can mimic other more common conditions like urinary tract infections or gastrointestinal issues [5]. The presented case involves a patient with cyclical abdominal pain for several months, a symptom typically associated with obstructive menstrual disorders. The diagnosis was delayed until she developed acute urinary retention (AUR), highlighting the importance of considering non-gynecological causes for abdominal pain. The prior use of indigenous medications may have contributed to this delay, which can lead to serious complications such as hydronephrosis. Similar studies have shown that women often experience misdiagnosis related to abdominal pain, resulting in delayed treatment for conditions like renal obstruction [6]. Studies have emphasized that early recognition and diagnosis of hematocolpos are essential to prevent long-term renal damage and recurrent infections [5]. Many authors emphasize the necessity for increased awareness among healthcare providers regarding the overlap between gynecological symptoms and renal issues [7]. This case illustrates the need for comprehensive evaluations in patients with ambiguous abdominal pain. Timely diagnosis is crucial to preventing adverse outcomes such as hydronephrosis.

Ultrasound remains the imaging modality of choice for diagnosing hematocolpos, as it provides a clear visualization of fluid accumulation in the vaginal and uterine cavities [8]. In this case, ultrasound confirmed the presence of a large hematocolpos. The findings on ultrasound were critical in guiding the decision for surgical intervention, which is the definitive treatment for hematocolpos caused by an imperforate hymen.

Surgical management, typically in the form of a hymenectomy, is curative for hematocolpos and provides immediate relief of symptoms, as demonstrated in this case. Post-operative outcomes are generally favorable, with most patients experiencing a complete resolution of urinary retention and a return to normal menstrual function [5]. In this patient, the hymenectomy allowed for the drainage of approximately 800 mL of retained menstrual blood, and her urinary and abdominal symptoms resolved shortly after the procedure. Follow-up care is essential to monitor for any post-operative complications and to provide education on menstrual health and hygiene, which is particularly important for adolescents who may be unfamiliar with normal menstrual patterns.

This case also highlights the psychosocial impact of delayed menarche and the associated physical symptoms on adolescents. It is important for healthcare providers to maintain open communication with both the patient and their family, addressing any concerns about the delay in menstruation and the potential need for surgical intervention. Early education on menstrual health and the importance of seeking medical care for abnormalities can prevent delays in diagnosis and treatment.

The limitations of this case include the absence of long-term follow-up data, which would provide insights into the patient's future gynecological health and potential recurrence of symptoms. Additionally, while this case report highlights the importance of considering gynecological causes in cases of AUR, larger studies are needed to better understand the prevalence and risk factors for hematocolpos presenting with urinary retention.

The work has been reported in line with the SCARE criteria [7].

## 4 Conclusion

In conclusion, this case reinforces the need for a high index of suspicion for gynecological conditions like hematocolpos in adolescent females presenting with urinary retention. Early diagnosis and prompt surgical intervention are crucial to prevent complications such as hydronephrosis and renal damage. Clinicians should be aware of this rare but significant cause of AUR, particularly in patients with a history of primary amenorrhea and cyclic abdominal pain.

## Author contribution

R.M: Writing - review & editing, Writing – original draft, Visualization, Validation, Supervision, Software, Resources, Project administration, Methodology, Funding acquisition, Formal analysis, Data curation, Conceptualization.

E.W.: Writing - review & editing, Writing – original draft.

E.M.: Writing - review & editing, Writing – original draft.

M.N.: Writing - review & editing, Writing – original draft.

J-L.M.: Writing - review & editing, Writing – original draft.

## Consent

Written informed consent was obtained from the patient's parents/legal guardian for publication and any accompanying images. A copy of the written consent is available for review by the Editor-in-Chief of this journal on request.

## Ethical approval

The Ethical Committee of the Hospital provided ethical approval for this study on 01 September 2024.

## Guarantor

Roland Muyisa, MD.

## Research registration number

1. Name of the registry: General Referral Hospital of Musienene.
2. Unique identifying number or registration ID: Not official registry.
3. Hyperlink to your specific registration (must be publicly accessible and will be checked): none.

## Funding

The authors declare that no funds, grants, or other support were received during the preparation of this manuscript.

## Conflict of interest statement

Authors have no conflict of interest to declare.
